# Supplementary figures and images for: Bayesian Decision Tree for the Classification of the Mode of Motion in Single-Molecule Trajectories
Source: PLoS One. 2013 Dec 20;8(12):e82799. doi: 10.1371/journal.pone.0082799 (PMC3869729; doi:10.1371/journal.pone.0082799)

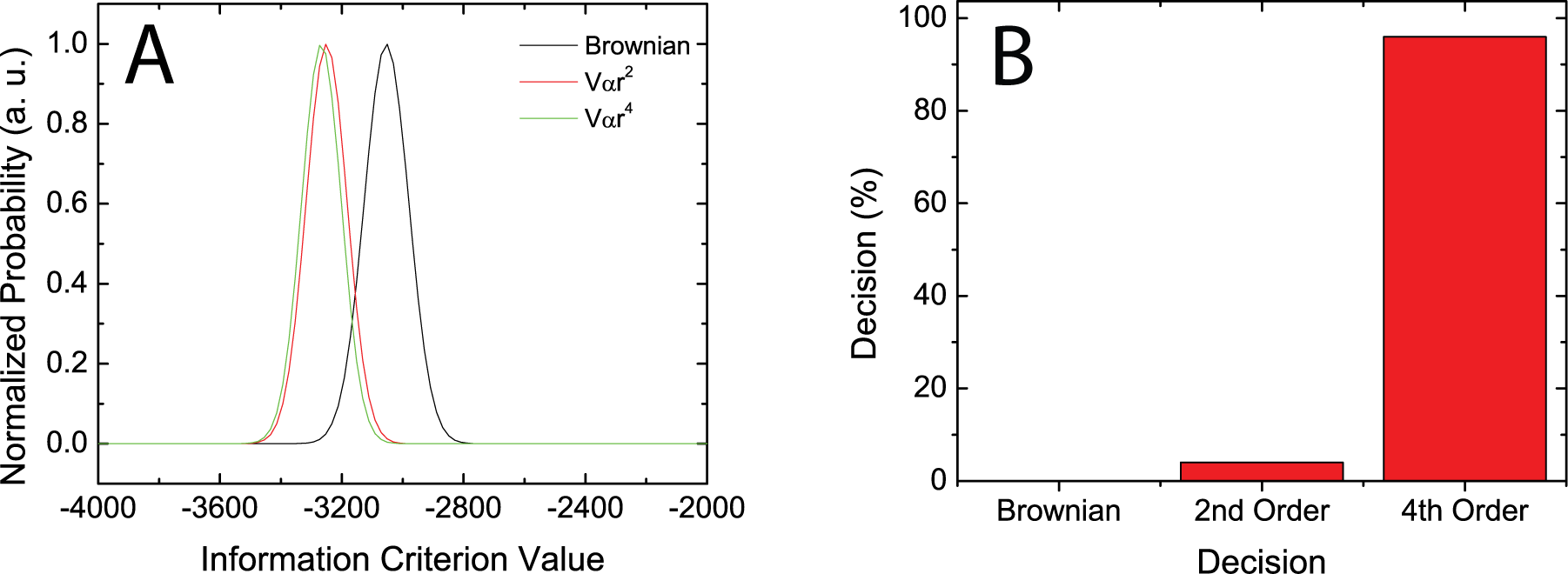

Supplement: Figure S1 — Averaged versus single-trajectory decision making. (A) Calculating and averaging the information theory criteria, such as the here displayed AIC, for a distribution of 100 numerical trajectories (Parameters: N = 500 points, shows that it is possible to distinguish between free Brownian motion and confined motion, but it is impossible to determine the nature of the confining potential from the averaged data. (B) However, making decisions based on the criteria for each individual trajectory can lead to a histogram that correctly identifies the input potential. (TIF) [file pone.0082799.s001.tif]

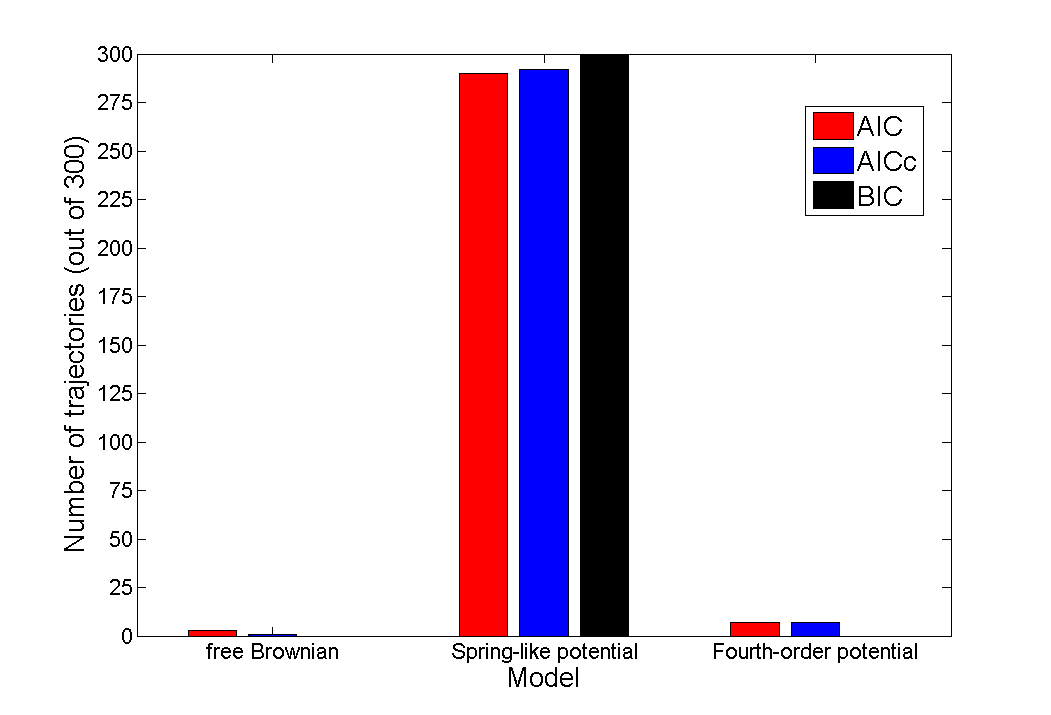

Supplement: Figure S2 — Histogram of trajectory classifications out of 300 numerical input trajectories that resemble experimental trajectories with confinement in a spring-like potential. (Parameters: N = 500 points, Decisions based on the BIC are shown in black, decisions based on the AIC and AICc are shown in red and blue, respectively. (TIF) [file pone.0082799.s002.tif]

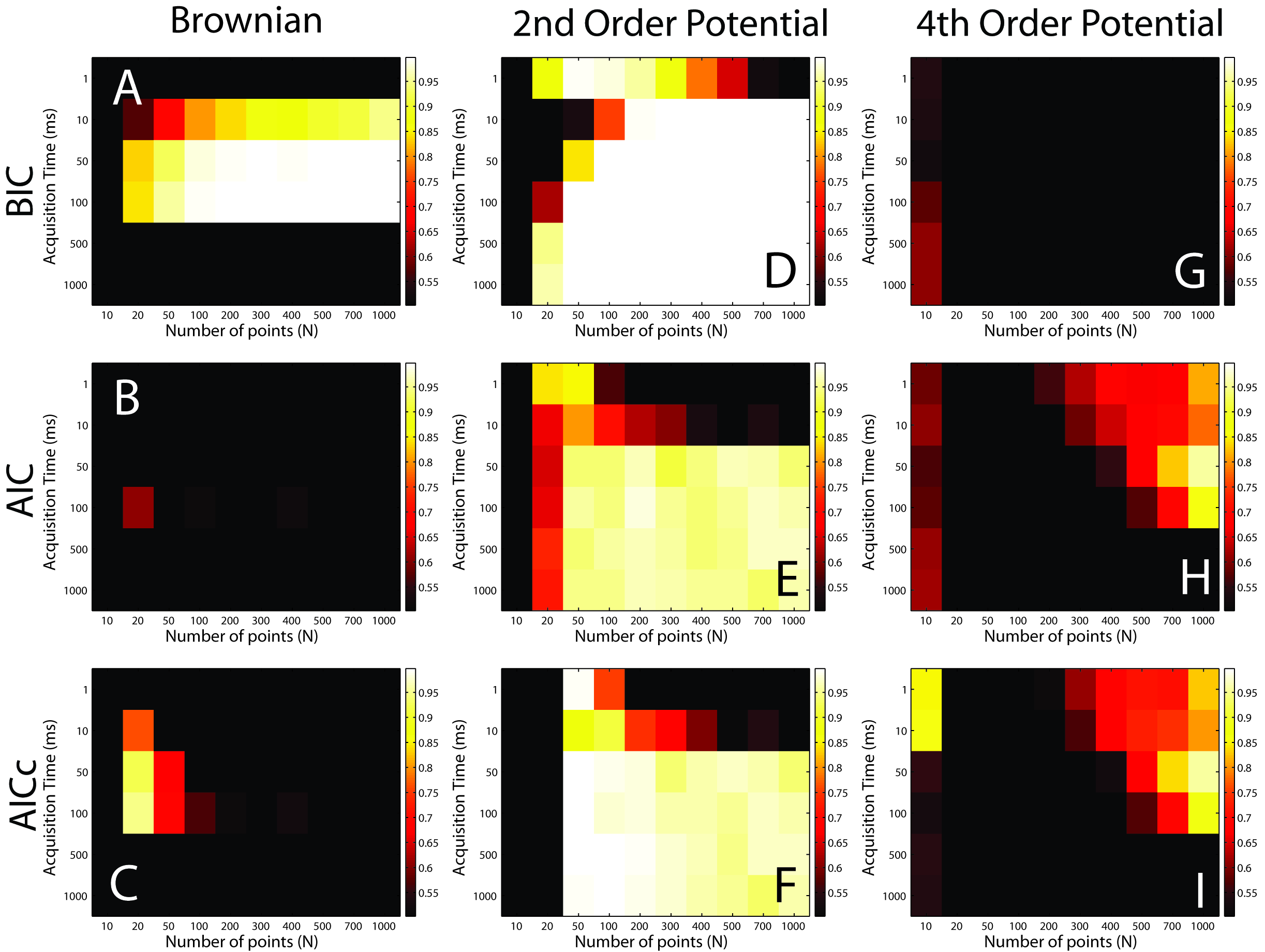

Supplement: Figure S3 — Building the decision tree using information criteria from simulated trajectories. The 2D plots show the heat map of the percentage of correct decisions out of 300 simulated trajectories per square for the BIC (first row), AIC (middle row), and AICc (bottom row). The input trajectories were Brownian (left column), confined in a spring potential (middle column), and confined in a 4th order potential (right column). The heat map has a threshold of 0.5, which means that only cases where the information criterion works correctly more than half of the time are non-black as indicated by the color scale. The BIC is the better criterion to determine if a trajectory is undergoing purely Brownian motion or if is confined by a potential (red box & red arm in decision tree in Fig. 1). The BIC is not suited to distinguish between a 2nd and 4th order potential. Here, the AIC and AICc provide a solution (blue box & blue arm in decision tree in Fig. 1). (TIF) [file pone.0082799.s003.tif]

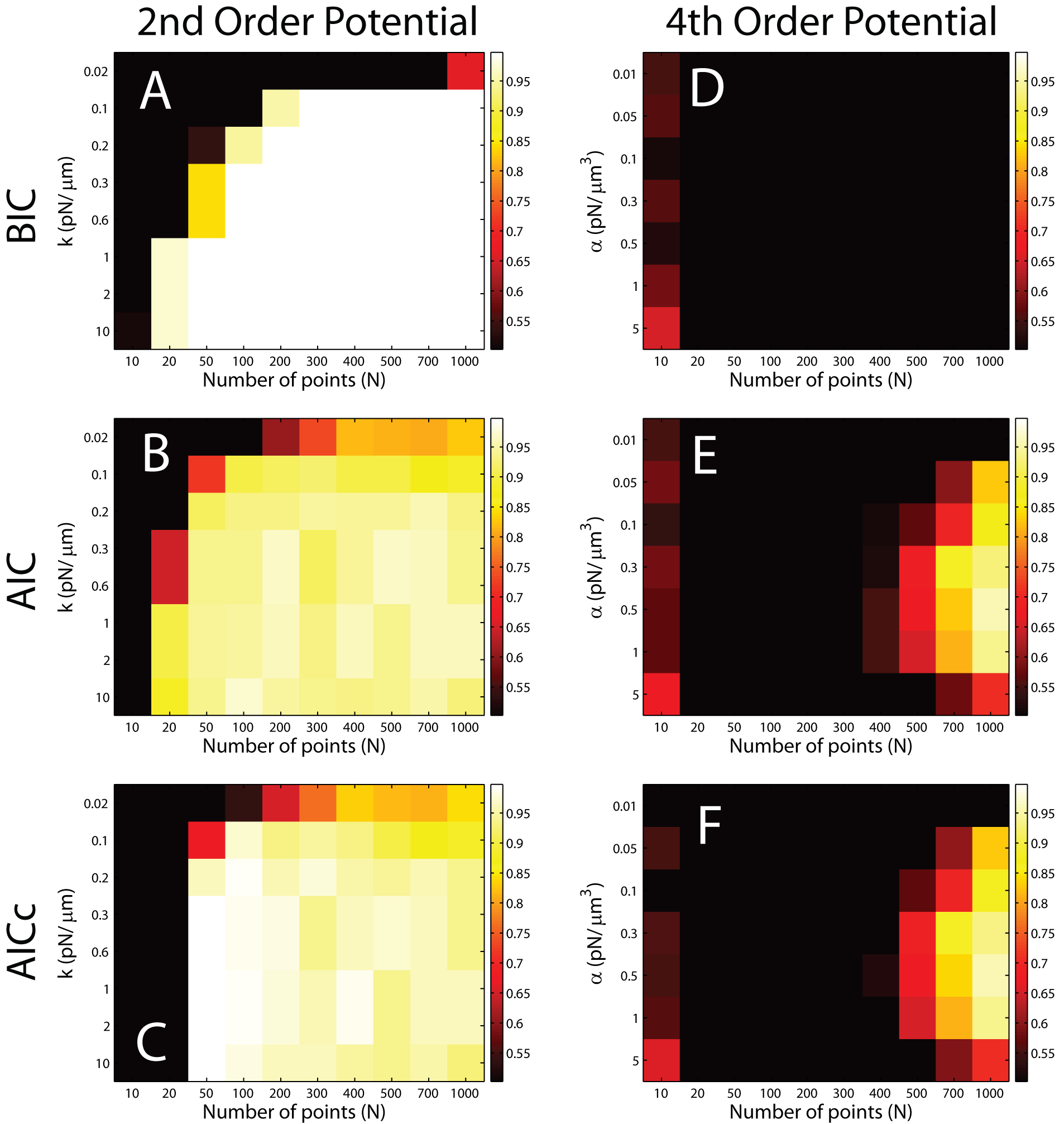

Supplement: Figure S4 — Building the decision tree using information criteria from simulated trajectories. The 2D plots show the heat map of the percentage of correct decisions out of 300 simulated trajectories per square for the BIC (first row), AIC (middle row), and AICc (bottom row). The input trajectories were Brownian walkers, confined in a spring potential (left column A–C), and confined in a 4th order potential (right column D–F). The heat map has a threshold of 0.5, which means that only cases where the information criterion works correctly more than half of the time are non-black as indicated by the color scale. The AIC and AICc are the only effective indicator that can distinguish between these two potential types. However, the strength of the potential does not have a large impact on the performance of these criteria. (TIF) [file pone.0082799.s004.tif]

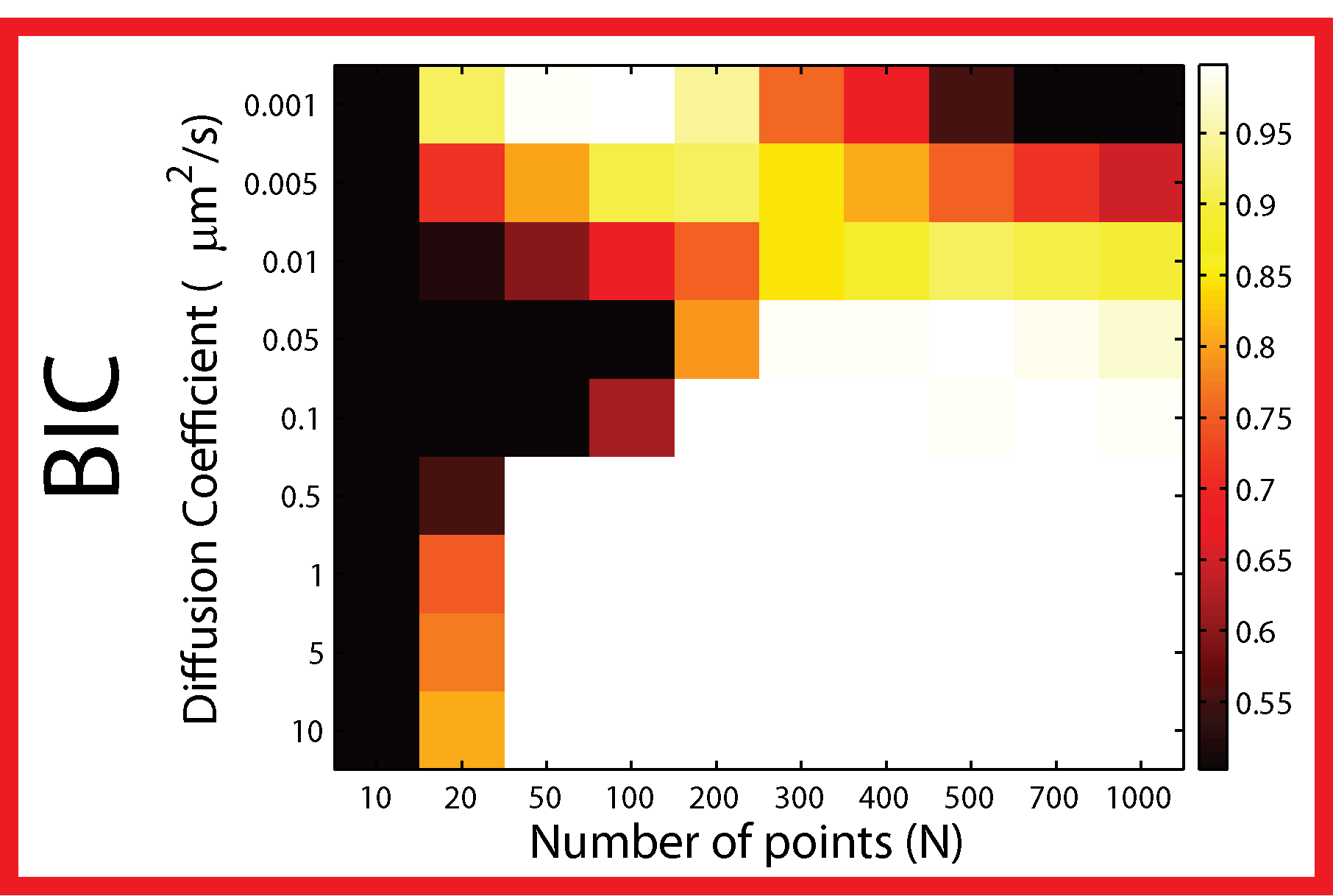

Supplement: Figure S5 — The BIC erroneously classifies the 4th order potentials to be 2nd order spring potentials. The 2D plot shows the heat map of the percentage of trajectories classified to have a 2nd order spring potential out of 300 simulated 4th order trajectories per square for the BIC. The heat map has a threshold of 0.5, which means that only cases where the information criterion falsely chooses 2nd order more than half of the time are non-black as indicated by the color scale. As mentioned earlier, the BIC cannot correctly attribute the 4th order potential, but finds a 2nd order spring-potential instead. Although this is clearly wrong, it can be exploited to build a two-step decision tree that can correctly distinguish all three cases using a mixture of BIC and AIC. (TIF) [file pone.0082799.s005.tif]

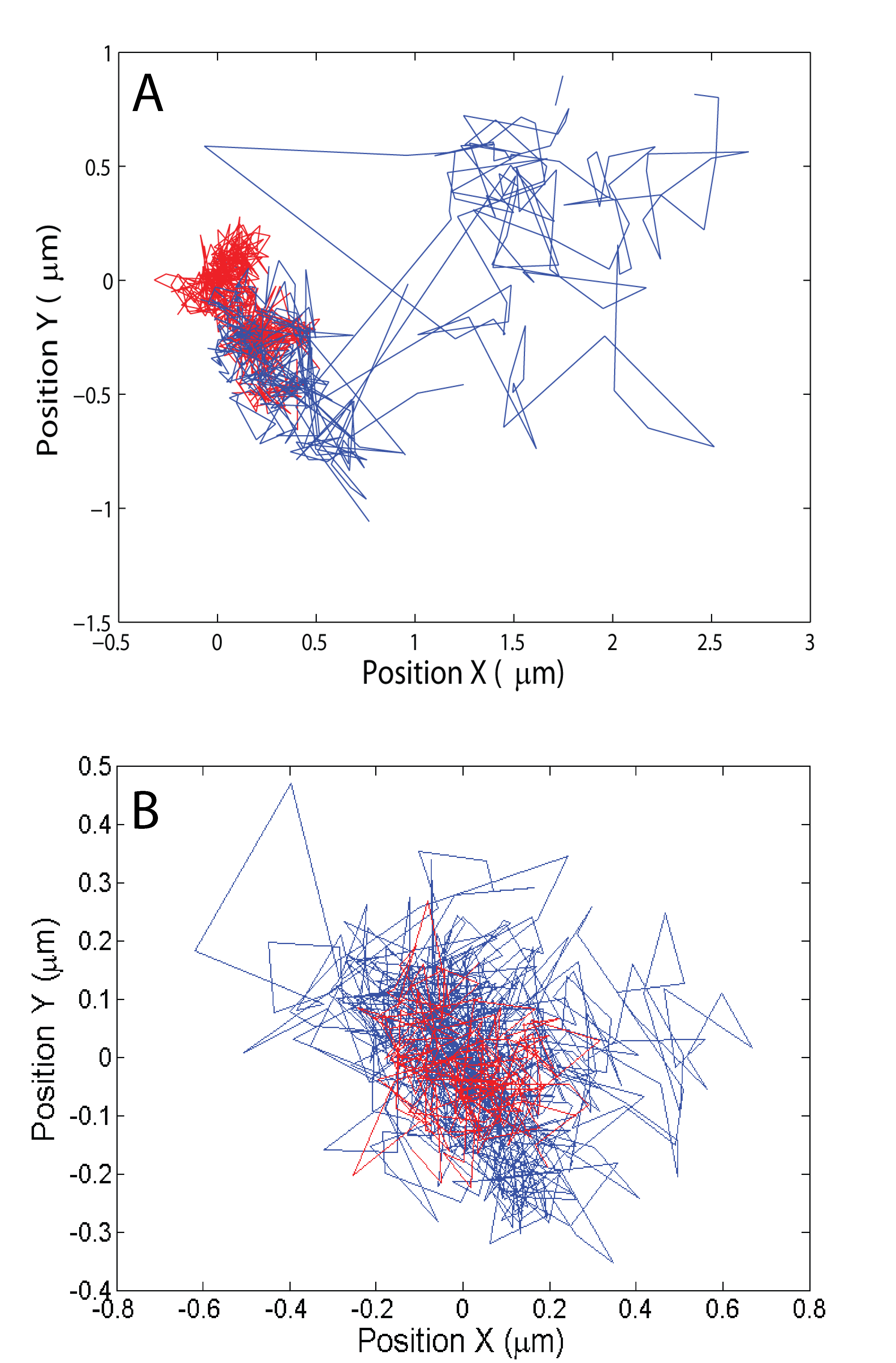

Supplement: Figure S6 — Inferring the mode of motion of single experimental CPT receptors during disaggregation of the confining domain. Two different trajectories are shown in A and B. We use the decision tree to determine the mode of motion on a 51 frame window that slides along the trajectory in time, while the cells are treated with cholesterol oxidase. The trajectory begins being confined by a spring-like potential (red). As the enzyme cholesterol oxidase oxidizes more cholesterol, the trajectory receptor becomes less confined and undergoes free Brownian motion (blue). (TIF) [file pone.0082799.s006.tif]

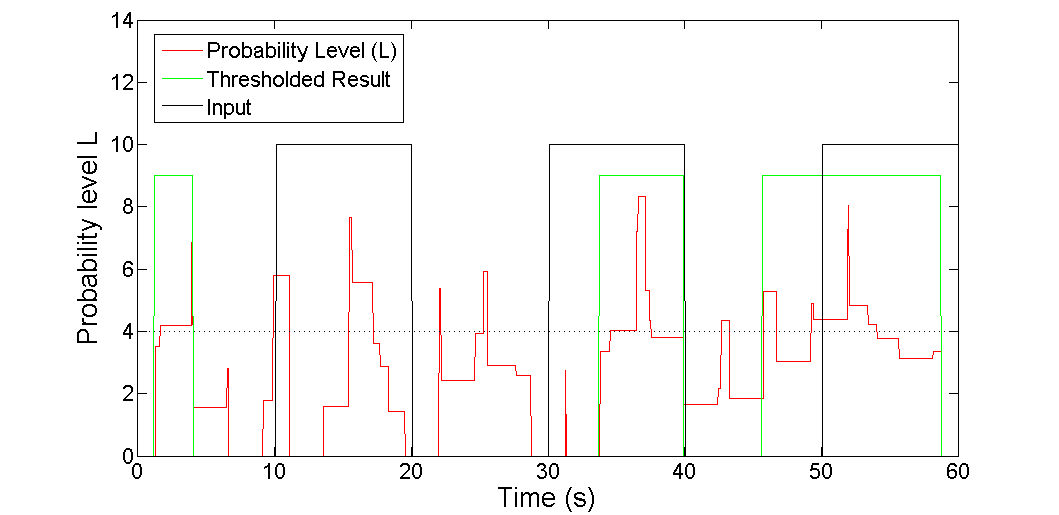

Supplement: Figure S7 — Residence time method for the detection of temporal lateral confinement. A single trajectory has three different confinement zones in a second order potential. (Parameters: The three confined parts of the trajectory are shown in black where a non-zero value indicates confinement. Outside of the confined zones, the trajectory undergoes free Brownian motion. The probability level (L) is shown in red along the trajectory. L is filtered in magnitude with a cutoff (dotted line) and temporal To qualify as a confinement zone, L has to be larger than for a time greater than The parameters were optimized to detect the confinement zones without generating many false positives. The threshold result displaying the confinement zones is shown in green. The method could correctly determine two out of three confining zones and found one false confinement zone (false-positive). (TIF) [file pone.0082799.s007.tif]
